# Beneficial effect of combinational methylprednisolone and remdesivir in hamster model of SARS-CoV-2 infection

**DOI:** 10.1080/22221751.2021.1885998

**Published:** 2021-02-25

**Authors:** Zi-Wei Ye, Shuofeng Yuan, Jasper Fuk-Woo Chan, Anna Jinxia Zhang, Ching-Yun Yu, Chon Phin Ong, Dong Yang, Chris Chun-Yiu Chan, Kaiming Tang, Jianli Cao, Vincent Kwok-Man Poon, Chris Chung-Sing Chan, Jian-Piao Cai, Hin Chu, Kwok-Yung Yuen, Dong-Yan Jin

**Affiliations:** a State Key Laboratory of Emerging Infectious Diseases and Department of Microbiology, The University of Hong Kong, Pokfulam, Hong Kong; b School of Biomedical Sciences, The University of Hong Kong, Pokfulam, Hong Kong

**Keywords:** COVID-19, SARS-CoV-2, remdesivir, corticosteroid, combination therapy

## Abstract

Effective treatments for coronavirus disease 2019 (COVID-19) caused by severe acute respiratory syndrome coronavirus 2 (SARS-CoV-2) are urgently needed. Dexamethasone has been shown to confer survival benefits to certain groups of hospitalized patients, but whether glucocorticoids such as dexamethasone and methylprednisolone should be used together with antivirals to prevent a boost of SARS-CoV-2 replication remains to be determined. Here, we show the beneficial effect of methylprednisolone alone and in combination with remdesivir in the hamster model of SARS-CoV-2 infection. Treatment with methylprednisolone boosted RNA replication of SARS-CoV-2 but suppressed viral induction of proinflammatory cytokines in human monocyte-derived macrophages. Although methylprednisolone monotherapy alleviated body weight loss as well as nasal and pulmonary inflammation, viral loads increased and antibody response against the receptor-binding domain of spike protein attenuated. In contrast, a combination of methylprednisolone with remdesivir not only prevented body weight loss and inflammation, but also dampened viral protein expression and viral loads. In addition, the suppressive effect of methylprednisolone on antibody response was alleviated in the presence of remdesivir. Thus, combinational anti-inflammatory and antiviral therapy might be an effective, safer and more versatile treatment option for COVID-19. These data support testing of the efficacy of a combination of methylprednisolone and remdesivir for the treatment of COVID-19 in randomized controlled clinical trials.

## Introduction

The ongoing pandemic of coronavirus disease 2019 (COVID-19) caused by infection with severe acute respiratory syndrome coronavirus 2 (SARS-CoV-2) has resulted in substantial morbidity and mortality globally [1]. With probable exception of certain areas in which herd immunity might have developed, most people around the world remain highly susceptible to SARS-CoV-2 [2,3]. While several types of safe and effective vaccines have been conditionally approved for human use [4–7], development of effective treatments to reduce severity and mortality of COVID-19 is another top priority. Most people infected with SARS-CoV-2 are asymptomatic or have mild symptoms [1,8,9]. If the mortality rate of COVID-19 can be reduced to those of seasonal influenza or common cold caused by community-acquired human coronaviruses, its devastating impact on global economy and people’s normal life would be substantially lessened.

Combination therapies consisting of drugs directed against both viral and host targets that govern different stages of the viral replication cycle and of the disease may have advantages over monotherapy targeting a single viral protein or enzyme. Our recent Phase 2 randomized clinical trial shows that triple combination of ribavirin, lopinavir-ritonavir and interferon β-1b alleviates symptoms, and shortens virus shedding and recovery time in hospitalized patients with mild to moderate COVID-19 [10]. Whereas ribavirin is a guanosine analog with broad-spectrum antiviral activities mediated through multiple mechanisms including inhibition of viral polymerase and mRNA capping, lopinavir and ritonavir are small-molecule inhibitors of viral proteases [11]. On the other hand, interferon β is a cytokine with antiviral and immunomodulatory activities [12]. Some patients under the triple therapy develop diarrhea and drug-induced hepatitis. Thus, additional safe and effective combination therapeutic options for COVID-19 are urgently needed.

Remdesivir is a nucleoside analog that represses SARS-CoV-2 replication by inhibiting viral RNA polymerase and the proofreading exoribonuclease [13]. Remdesivir is not only efficacious for the treatment of SARS-CoV-2 infection in cellular and mouse models [14], but also confers the benefit of a shortened recovery time in human adult patients hospitalized with COVID-19 [15]. However, the benefits of remdesivir were only seen when it was given before and near the onset of disease and would progressively decrease or disappear if administered at the late phase of infection [16,17]. Surprisingly, the use of dexamethasone, a glucocorticoid with immuno-suppressive and anti-inflammatory properties [18], has recently been shown to reduce 28-day mortality in British patients hospitalized with COVID-19 and requiring respiratory support [19]. Similar beneficial effect of dexamethasone or methylprednisolone has also been observed in Brazilian patients hospitalized with COVID-19 who were either suffering from moderate to severe acute respiratory distress syndrome [20] or at the age of more than 60 years-old [21]. Consistent with this trend, among Chinese COVID-19 patients with marked radiological progression, use of glucocorticoids for a short term and at low to moderate dose reduced the need for invasive mechanical ventilation only in a group at an early phase of excessive inflammation with a lactose dehydrogenase level of less than two times the upper limit of normal [22]. Since COVID-19 is an immune-mediated inflammatory disease caused by SARS-CoV-2 [1,23], both reduction of viral loads and alleviation of inflammation are desired in its treatment. The administration of glucocorticoids is known to sensitize macrophages and mice to infection with mouse hepatitis virus (MHV), a prototypic coronavirus [24,25]. Deleterious effects are also reported in the treatment of SARS and Middle East respiratory syndrome with glucocorticoids [26,27]. The possibility that a glucocorticoid could stimulate SARS-CoV-2 replication under some circumstances cannot be underestimated. To eliminate the proviral effect of glucocorticoid, its combination with an effective antiviral such as remdesivir should be considered. Thus, we set out to assess the effect of methylprednisolone alone and its combination with remdesivir in the established hamster model of SARS-CoV-2 infection.

## Materials and methods

### Chemicals, viruses and cells

Methylprednisolone was purchased from Pfizer and was prepared as per manufacturer’s instruction. Remdesivir was purchased from MedChemExpress (Monmouth Junction, NJ, USA). SARS-CoV-2 virus HKU-001a strain (GenBank accession number: MT230904) was isolated from the nasopharyngeal aspirate specimen of a laboratory-confirmed COVID-19 patient in Hong Kong, and was cultured and titrated in Vero-E6 cells by plaque assays. All experiments involving live SARS-CoV-2 were performed in a Biosafety Level-3 facility.

Peripheral blood samples from healthy volunteers were acquired from Hong Kong Red Cross Blood Transfusion Service according to a protocol approved by the Joint Institutional Review Board of the University of Hong Kong and the West Cluster of the Hospital Authority of Hong Kong. Human peripheral blood mononuclear cells (PBMCs) were isolated from the buffy coats as previously described [28]. MDMs were differentiated from PBMCs by providing RPMI-1640 supplemented with 10% FBS, 1% sodium pyruvate, 1% non-essential amino acids, 100 units/mL of penicillin, 100 μg/mL of streptomycin and 1 U/mL of granulocyte-macrophage colony stimulating factor (Cell Sciences, Canton, MA, USA) for 6–7 days before infection.

### Virus infection and methylprednisolone treatment

**Figure 1.**
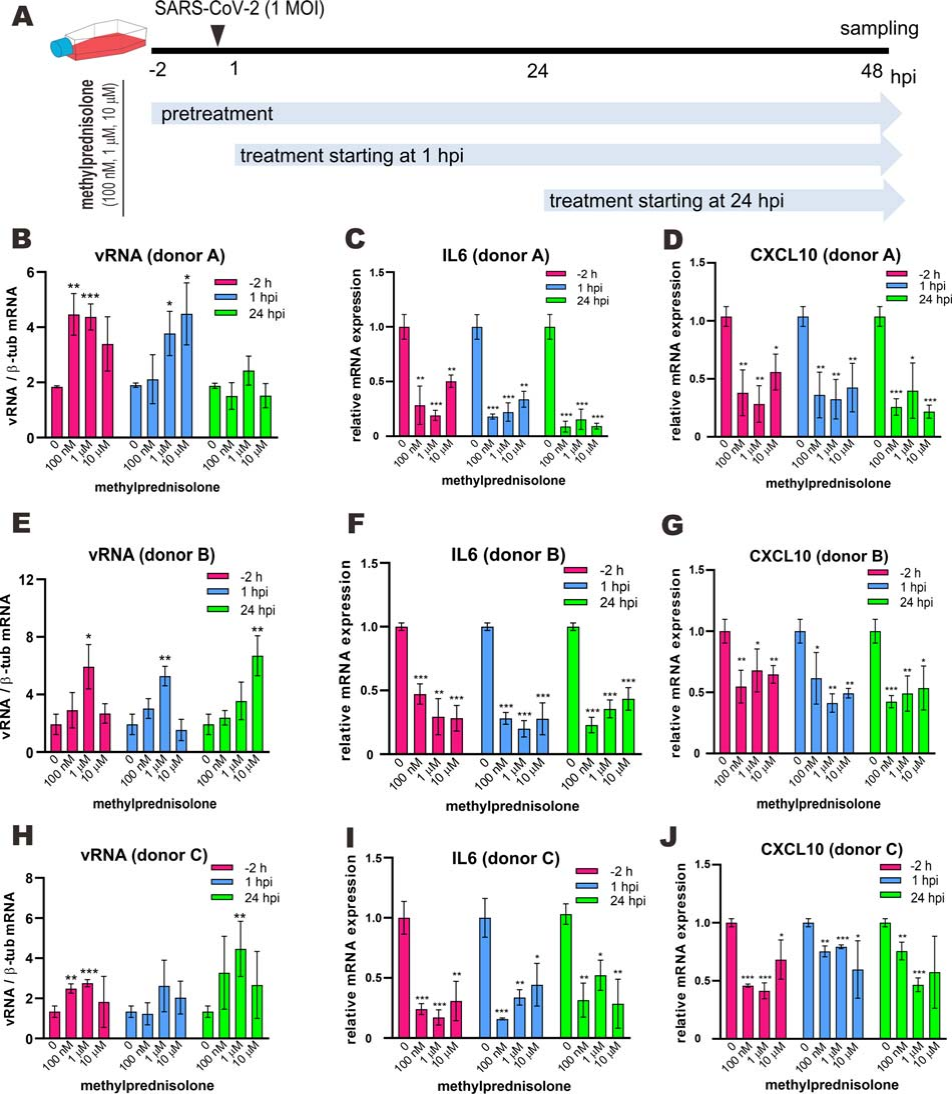
Effect of methylprednisolone on SARS-CoV-2 RNA replication and viral induction of cytokine expression in monocyte-derived macrophages. (A) Treatment scheme indicating different time points and doses of methylprednisolone addition before or after SARS-CoV-2 infection at 1 MOI in monocyte-derived macrophages from multiple donors. (B–J) Intracellular viral RNA loads and viral induction of the expression of selected proinflammatory cytokines. Cell lysates were collected at 48 hpi and viral genome copies were detected by RT-qPCR (B, E and H). Relative expression of IL6 (C, F and I) and CXCL10 (D, G and J) was examined by RT-qPCR. Results are presented after normalization to human β-tubulin (β-tub) transcript. Difference between the indicated group and the no treatment group was statistically significant as judged by Student’s *t* test (*P < 0.05; **P < 0.01; ***P < 0.001).

Human MDMs were infected with SARS-CoV-2 at an MOI of 0.1 or 1 for 2 h at 37 °C. After a 2-hour incubation, the virus inoculum was washed off and the cells were maintained with fresh RPMI-1640. As shown in Figure 1(A), three different doses of methylprednisolone (100 nM, 1 μM or 10 μM) were added into the culture medium 2 h before SARS-CoV-2 infection, 1 h post-infection (hpi) and 24 hpi. At 48 hpi, cell culture supernatant and lysates were collected for viral load analysis.

### Infection of golden Syrian hamsters

All experimental protocols were approved by the Committee on the Use of Live Animals in Teaching and Research (CULATR) of the University of Hong Kong and were performed according to the standard operating procedures of the biosafety level 3 animal facilities (Reference code: CULATR 5370-20) in adherence to the NIH Guide for Care and Use of Laboratory Animals. Animal infection experiments were performed as we previously described with slight modifications [29,30]. Male and female golden Syrian hamsters, aged 6–8 weeks old, were obtained from the Chinese University of Hong Kong Laboratory Animal Service through the Center for Comparative Medicine Research of the University of Hong Kong. The animals were raised in Biosafety Level-2 facility with access to standard pellet feed and water *ad libitum*. Baseline body weights were recorded before infection. PBS was used to dilute virus stocks to the desired concentration. Under anesthesia with intraperitoneal injection of ketamine (200 mg/kg) and xylazine (10 mg/kg), a challenge dose of 10^5^ plaque forming units (PFU) of SARS-CoV-2 was inoculated intranasally to each hamster. In steroid treatment groups, hamsters were intraperitoneally or intramuscularly injected with methylprednisolone (10 mg/kg) at 2 dpi or 5 dpi. For the drug combination group, hamsters were intraperitoneally injected with remdesivir (15 mg/kg) at 2 dpi and 3 dpi. Remdesivir was prepared as 100 mg/ml stock in dimethyl sulfoxide and further diluted using 12% sulfobutylether-β-cyclodextrin before intraperitoneal injection. Body weights were monitored daily for 14 days after infection. For virological and histopathological examinations, 3–5 hamsters per group were sacrificed at 4 dpi, 7 dpi or 14 dpi, and their organs (nasal turbinates, tracheas, lungs and blood) were collected for analyses. Half of the harvested tissues was used for virus titration by RT-qPCR method, while the other half was immediately fixed in 10% of PBS-buffered formaldehyde for histopathological analyses as described previously [29]. For histopathological assessment, slides were examined in a blinded manner and scored with a semi-quantitative system according to the relative degree of inflammation and tissue damage.

### Titer of anti-receptor-binding domain (RBD) antibodies in hamster sera

Enzyme immunoassay (EIA) was performed as described [31]. Ninety-six-well plates (Nunc, Rochester, NY, USA) were coated with 0.1 μg/well of SARS-CoV-2 RBD in 100 μl of 0.05 M NaHCO_3_ (pH 9.6) overnight at 4°C. After blocking, 100 μl of heat-inactivated serum samples were serially diluted before adding to the wells and incubated at 37°C for 1 h. The attached antibodies were detected using horseradish-peroxidase-conjugated rabbit anti-hamster IgG antibody (A18895 from Invitrogen, Waltham, MA, USA), followed by addition of diluted 3,3′,5,5′-tetramethylbenzidine single solution (Invitrogen) and 0.3 N H_2_SO_4_. The optical density at 450 nm (OD_450_) was read using a microplate reader.

### RT-qPCR

RNA extraction, reverse transcription, and qPCR were performed as previously described [28,32]. Briefly, total RNA was extracted from hamster lungs, nasal turbinates or tracheas with RNeasy Mini Kit (Qiagen, Germantown, MD, USA) and reverse transcribed with Transcriptor First Strand cDNA Synthesis Kit (Roche, Basel, Switzerland). Real-time PCR was performed using StepOne Plus Real-Time PCR System (Applied Biosystems, Forster City, CA, USA) according to the manufacturer’s instructions. Relative gene expression was normalized to the corresponding β-actin or GAPDH values.

### Immunostaining and confocal imaging

Immunostaining was performed to visualize SARS-CoV-2 N protein in hamster lung tissues as previously described [31]. In-house rabbit antiserum against SARS-CoV-2 N protein and goat anti-rabbit Alexa Fluor (Thermo Fisher Scientific, Waltham, MA, USA) were used as primary and secondary antibodies, respectively. The nuclei of cell were stained by 4′,6-diamidino-2-phenylindole (DAPI) nucleic acid stain from Thermo Fisher Scientific. Images were acquired with a Carl Zeiss (Dublin, CA, USA) LSM880 system.

## Results

### Promotion of SARS-CoV-2 RNA replication in human macrophages by methylprednisolone

Suppression of immune response by glucocorticoids has the risk of boosting viral replication. Indeed, upon treatment with cortisone acetate, genetically resistant mice and liver macrophages from these mice become susceptible to infection with MHV [24]. Administration of methylprednisolone exacerbates histopathological changes in the liver of MHV-infected mice and increases viral loads by 50-fold to more than 1000-fold. The deleterious effect can be observed with as little as 0.1 mg methylprednisolone per day [25]. To test how methylprednisolone might affect SARS-CoV-2 infection of human cells, monocyte-derived macrophages (MDMs) prepared from multiple donors were used. MDMs were chosen since immune cells are the primary targets of glucocorticoids [16] and the primary source of cytokines during SARS-CoV-2 infection [1]. MDMs have previously been shown to be capable of supporting abortive infection of SARS-CoV-2 [33,34]. The MDMs from three donors were pretreated with three different doses of methylprednisolone 2 h before infection with SARS-CoV-2 at an MOI of 1. The hormone was also separately given at an early time point of 1 hpi and another late time point of 24 hpi (Figure 1(A)). Whereas donor-to-donor difference in the response of SARS-CoV-2 infection to methylprednisolone was noticed, boosting effects of 2- to 3-fold on viral RNA loads measured by RT-qPCR analysis of viral RNA were consistently observed in MDMs derived from all three donors (Figure 1(B, E, H)). In keeping with this trend, methylprednisolone also augmented SARS-CoV-2 RNA loads to different extents in MDMs derived from three other donors and infected with SARS-CoV-2 at an MOI of 0.1 (Figure S1(B–J)). Concurrently, consistent with the immunosuppressive effect of methylprednisolone, induction of representative proinflammatory cytokines interleukin 6 (IL6) and CXCL10 in MDMs infected with 1 MOI of SARS-CoV-2 was dampened by all three doses of methylprednisolone (Figure 1(C, F, I, D, G, J)). Thus, glucocorticoids have the potential to promote RNA replication of SARS-CoV-2 but suppress viral induction of proinflammatory cytokines in human cells.

**Figure 2.**
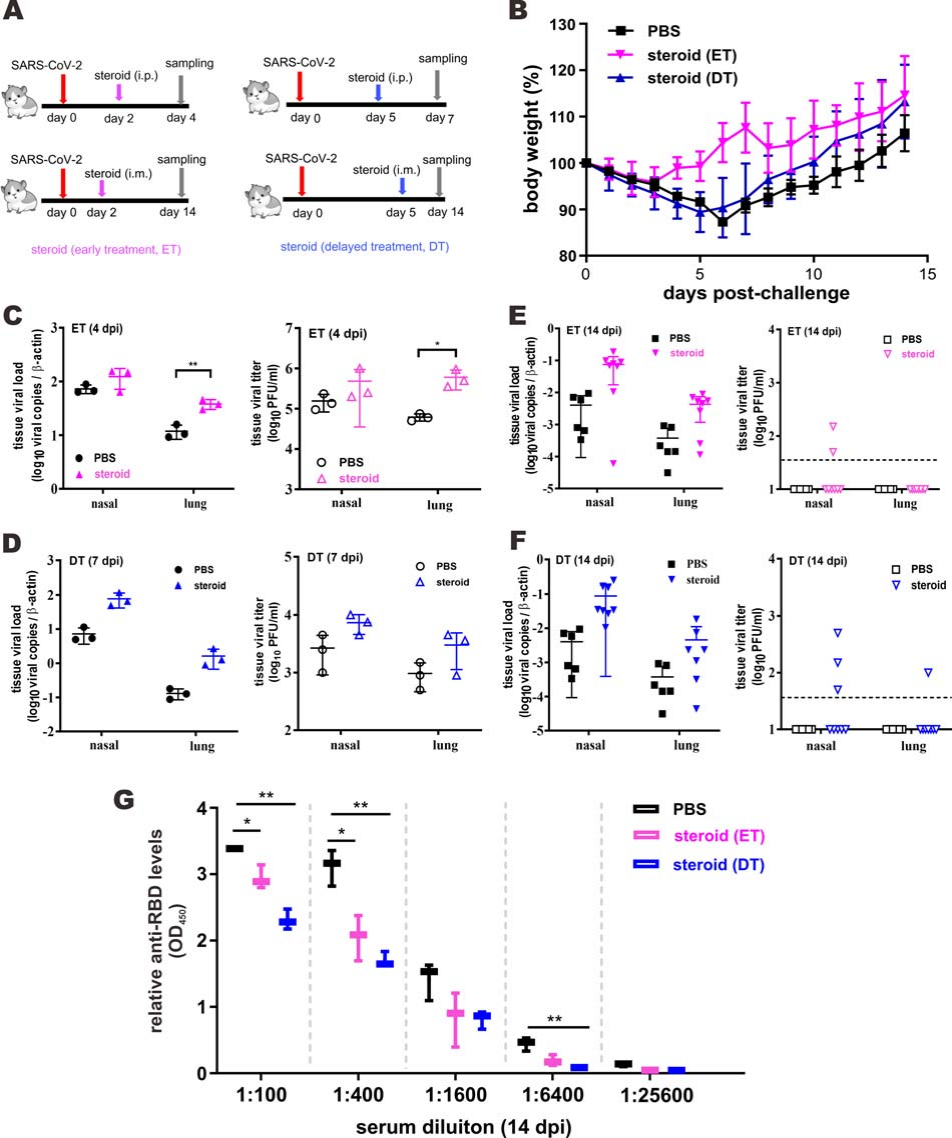
Beneficial effect of methylprednisolone in SARS-CoV-2-infected hamsters. (A) Diagram of the virus challenge scheme. Hamsters (*n* = 3) were intranasally inoculated with 10^5^ PFU of SARS-CoV-2 and were intraperitoneally (i.p.) given either methylprednisolone (10 mg/kg) or vehicle control (PBS) at 2 dpi (early steroid treatment, ET, pink) or 5 dpi (delayed steroid treatment, DT, blue) for rapid analysis of the impact on viral loads. At 4 dpi or 7 dpi (i.e. 2 days after methylprednisolone injection), respiratory tissue viral yields in the nasal turbinates and lung tissues of the hamsters were detected by RT-qPCR and plaque assay. Alternatively, infected hamsters (*n* ≥ 3) were intramuscularly (i.m.) injected with either methylprednisolone (10 mg/kg) or PBS at 2 dpi (ET in pink) or 5 dpi (DT in blue) for delayed analysis of the impact on viral loads at 14 dpi. (B) Dynamic changes of body weights of SARS-CoV-2-infected hamsters intramuscularly administered with PBS or methylprednisolone (ET and DT). (C, D) Viral loads by RT-qPCR and plaque assay in the nasal turbinates and lung tissues collected at 4 dpi and 7 dpi from SARS-CoV-2-challenged hamsters intraperitoneally administered with either PBS or methylprednisolone at 2 dpi (ET) and 5 dpi (DT), respectively. (E, F) Viral loads in the nasal turbinates and lung tissues collected at 14 dpi from SARS-CoV-2-challenged hamsters intramuscularly injected at 2 dpi (ET) or 5 dpi (DT). (G) EIA analysis of anti-RBD antibodies in serum samples harvested at 14 dpi from hamsters intramuscularly administered with PBS and steroid (ET and DT).

**Figure 3.**
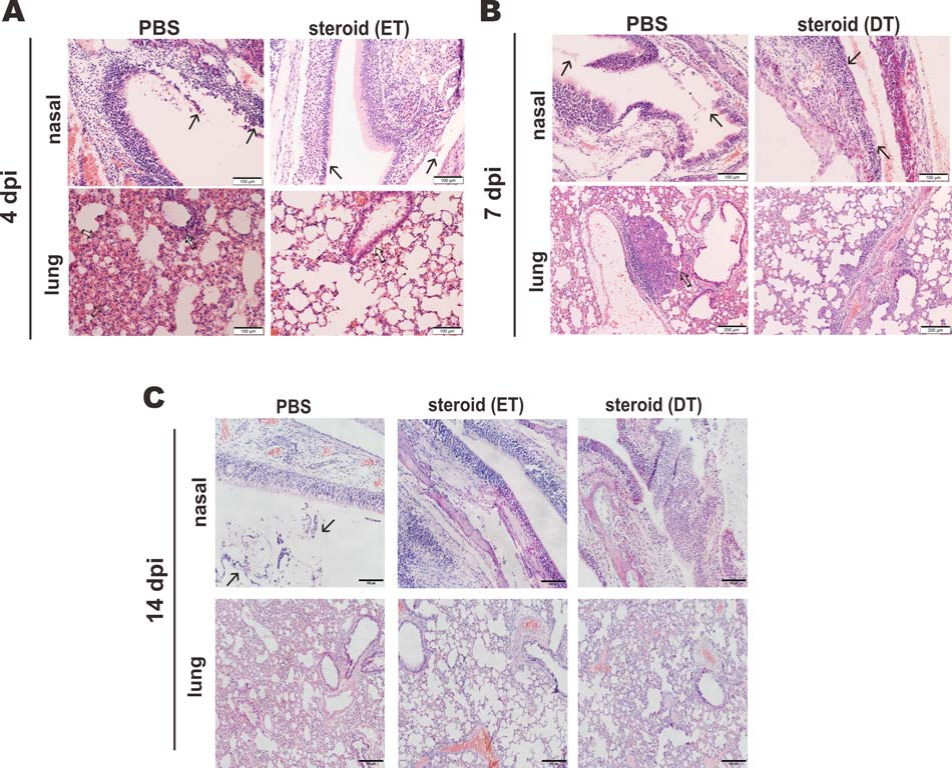
Impact of steroid on the histopathological changes in nasal turbinates and lungs of SARS-CoV-2-infected hamsters. Representative images of nasal turbinate and lung sections of SARS-CoV-2-infected hamsters were shown. Hamsters were intranasally inoculated with 10^5^ PFU of SARS-CoV-2 and then treated with methylprednisolone at 2 dpi (early steroid treatment, ET) or 5 dpi (delayed steroid treatment, DT). Histopathological changes in nasal turbinate and lung tissues were examined by hematoxylin and eosin staining. (A) Images at 4 dpi. Nasal turbinate tissue in PBS treatment control hamster showed severe epithelial desquamation and submucosal infiltration (arrows). The lung tissue showed diffuse alveolar wall thickening, blood vessel congestion, patchy area of alveolar space infiltration, exudation and patchy area of lung consolidation with two adjacent blood vessels showing vasculitis (open arrows). In ET group, respiratory and olfactory epithelium was intact, with mild submucosal infiltration observed in respiratory epithelium (arrows). In the lung tissue, only peribronchiolar infiltration and patchy area of alveolar wall thickening were observed (open arrows). Scale bar, 100 μm. (B) Images at 7 dpi showing histopathological changes in nasal turbinate and lung tissues from hamster receiving DT at 5 dpi. Nasal turbinate of the PBS control still showed some exudation mixed with cell debris in the nasal cavity (arrows), while the nasal epithelium showed prominent cell proliferation in steroid-treated hamster indicating tissue repairment (lower, arrows). Scale bar, 100 μm. The lung of PBS control hamster showed focal hemorrhage and patchy proliferative consolidation (open arrows); while in steroid-treated hamster, the lung still had mild perivascular infiltration but only very small foci of cell proliferation (open arrows). Scale bar, 200 μm. (C) Images at 14 dpi. Nasal turbinate tissue in PBS control hamster still showed mild degree intra-epithelium infiltration and submucosal blood vessel congestion. Luminal secretion with cell debris was occasionally observed (arrows). While nasal turbinate tissues showed intact epithelial layers, no apparent immune cell infiltration or luminal secretion was seen. Scale bar, 100 μm. The lung tissue in PBS control showed diffuse alveolar wall thickening and blood vessel congestion with no alveolar space infiltration or exudation. Upon early (ET) or delayed (DT) steroid treatment, the lung tissue showed only focal area of mild alveolar wall thickening and vessel congestion. Scale bar, 200 μm.

**Figure 4.**
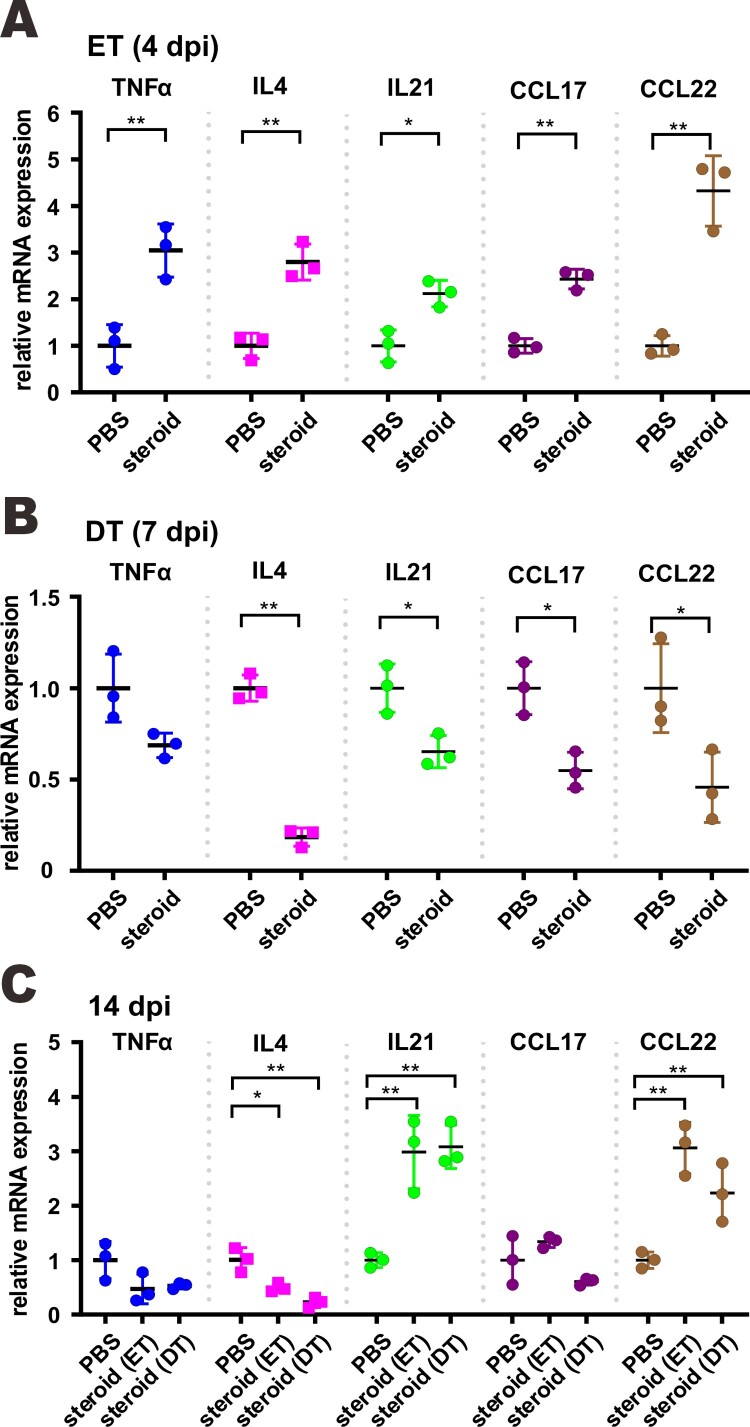
Proinflammatory cytokine and chemokine gene expression in SARS-CoV-2-infected hamster lung upon methylprednisolone treatment. Transcripts of representative chemokines and cytokines in the lung tissue homogenates of the indicated groups were detected by RT-qPCR at three time points. Results are shown as means ± SD. Difference between the indicated groups was statistically significant as judged by Student’s t test (*P < 0.05; **P < 0.01; ***P < 0.001).

*Beneficial effect of methylprednisolone monotherapy in SARS-CoV-2-infected hamsters.* Golden Syrian hamsters infected with SARS-CoV-2 represent a good small animal model in which symptoms and pathological changes similar to those of mild to moderate COVID-19 in humans can be seen [29,35]. The beneficial effect of dexamethasone and methylprednisolone was observable only in certain groups of COVID-19 patients but not universally [19–22]. This and the potential risk of glucocorticoids in sensitizing human cells to SARS-CoV-2 and boosting viral RNA replication (Figure 1) prompted us to examine the effect of methylprednisolone in SARS-CoV-2-infected hamsters more systematically. Since pulse glucocorticoid therapy has also been used successfully in the treatment of COVID-19 [36], we tested the effect of a single-dose methylprednisolone given intraperitoneally at an early time point of 2 dpi (early treatment, ET) or a late time point of 5 dpi (delayed treatment, DT) to hamsters intranasally infected with a high-dose of SARS-CoV-2 (Figure 2(A)). Tissue viral titers in the ET and DT groups were determined at 4 dpi or 7 dpi, respectively. Alternatively, infected hamsters were also intramuscularly administered with methylprednisolone for delayed analysis of the impact on viral loads and body weights. Intramuscular injection with methylprednisolone alone in the ET group resulted in the reversal of body weight loss (Figure 2(B)). A less prominent alleviating effect of methylprednisolone on body weight loss was also seen in the DT group, particularly at later time points (Figure 2(B)). However, viral RNA loads and viral titers in the nasal turbinates and lungs, as respectively measured by RT-qPCR and plaque assay, further increased when compared with the PBS control group at 2 days after steroid treatment (Figure 2(C, D)). In addition, viral RNA loads at 14 dpi were low but detectable in steroid treatment groups, indicating a delayed clearance of viral RNA, particularly from upper respiratory tract (Figure 2(E, F)). Importantly, serum titers of IgG antibodies against RBD of SARS-CoV-2 spike (S) protein were significantly lower in the ET and DT groups compared to those in the PBS control group (Figure 2(G)). Notably, histopathological examination of nasal turbinate and lung tissues at 2 days after steroid treatment confirmed that tissue damages and inflammation seen in PBS-treated hamsters, including alveolar wall thickening, blood vessel congestion, infiltration of immune cells in the alveolar space, exudation and vasculitis, were substantially relieved in animals receiving early or delayed treatment with methylprednisolone (Figure 3(A, B)). Consistent with this, inflammatory cell infiltration was also alleviated in steroid treatment groups at 14 dpi (Figure 3(C)). Detection of the transcripts of selected proinflammatory cytokines and chemokines including tumour necrosis factor α (TNFα), interleukin 4 (IL4), IL21 as well as C–C chemokines CCL17 and CCL22 indicated that their levels continued to increase further 2 days after early steroid treatment (Figure 4(A)) but decreased 2 days after delayed steroid treatment (Figure 4(B)). At 14 dpi, the levels of most cytokines and chemokines were comparably low between PBS and steroid treatment groups, with the exception of IL21 and CCL22, the levels of which remained high in the steroid groups (Figure 4(C)). Collectively, these results were generally in keeping with alleviation of inflammation with concurrent augmentation of SARS-CoV-2 replication by methylprednisolone monotherapy.

### Antiviral and anti-inflammatory effects of combinational methylprednisolone and remdesivir

**Figure 5.**
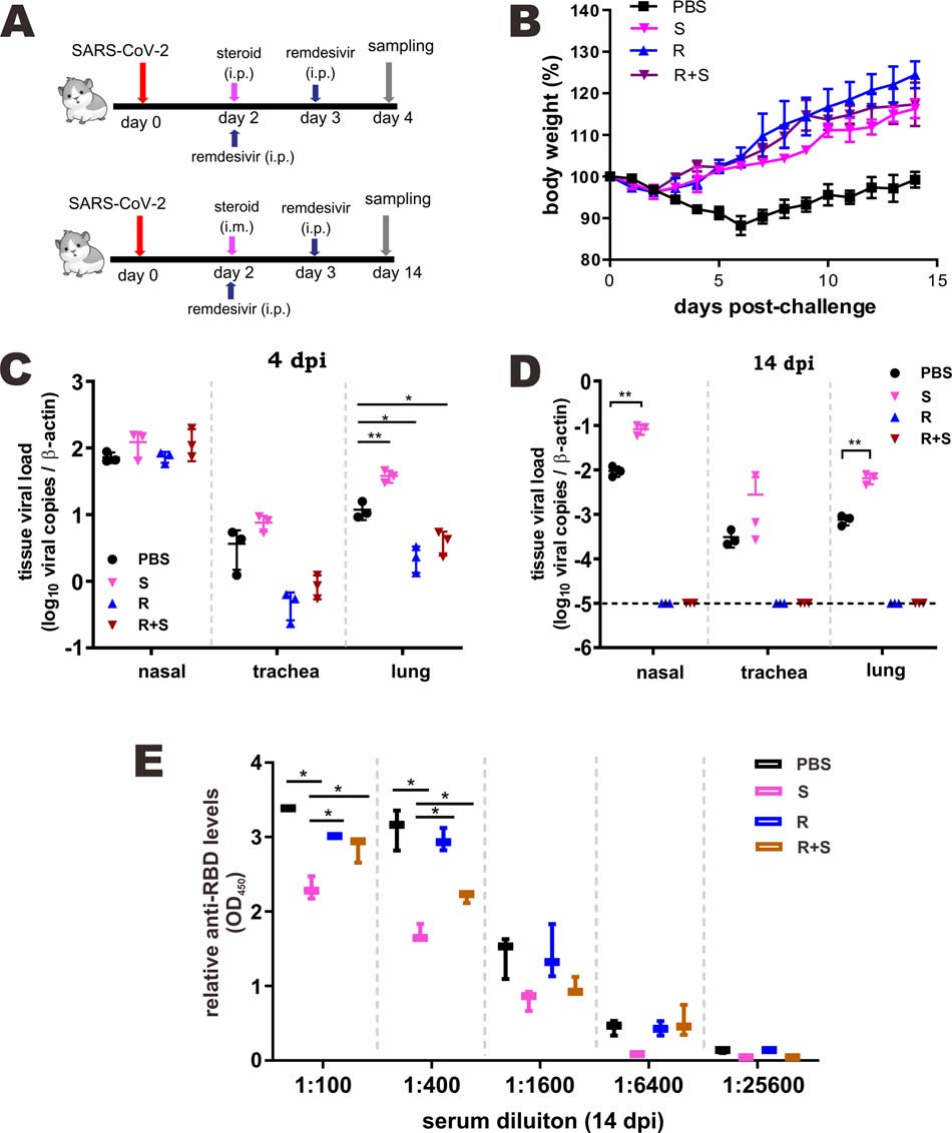
Effect of a combination of methylprednisolone and remdesivir in SARS-CoV-2-infected hamsters. (A) Diagram of the virus challenge scheme. Hamsters (*n* = 3) were intranasally inoculated with 10^5^ PFU of SARS-CoV-2 and then intraperitoneally administered with either methylprednisolone (10 mg/kg) or PBS at 2 dpi. At 2 dpi and 3 dpi post-challenge, hamsters were intraperitoneally (i.p.) injected with remdesivir (15 mg/kg). At 4 dpi (i.e. 2 days after methylprednisolone injection), viral yields in the nasal turbinates, lung tissues and tracheas of the hamsters were detected by RT-qPCR and plaque assay. Alternatively, infected hamsters (*n* ≥ 3) were intramuscularly (i.m.) given either methylprednisolone (10 mg/kg) or PBS at 2 dpi. At 2 dpi and 3 dpi, hamsters were intraperitoneally injected with remdesivir (15 mg/kg). Viral loads were analyzed at 14 dpi. (B) Body weight changes of SARS-CoV-2-challenged hamsters (*n* = 6 at 0 dpi to 4 dpi; *n* = 3 at 5 dpi to 14 dpi). S: steroid only. R: remdesivir only. R + S: remdesivir plus steroid. Steroid was given intramuscularly. (C, D) Viral loads by RT-qPCR and plaque assay in the nasal turbinates, lungs and tracheas of SARS-CoV-2-challenged hamsters at 4 dpi and 14 dpi (*n* = 3/group). Difference between the indicated groups was statistically significant as judged by Student’s t test (*P < 0.05; **P < 0.01). Steroid was given intraperitoneally (C) and intramuscularly (D). (F) EIA analysis of anti-RBD antibodies in sera of treated mice at 14 dpi. Steroid was given intramuscularly.

**Figure 6.**
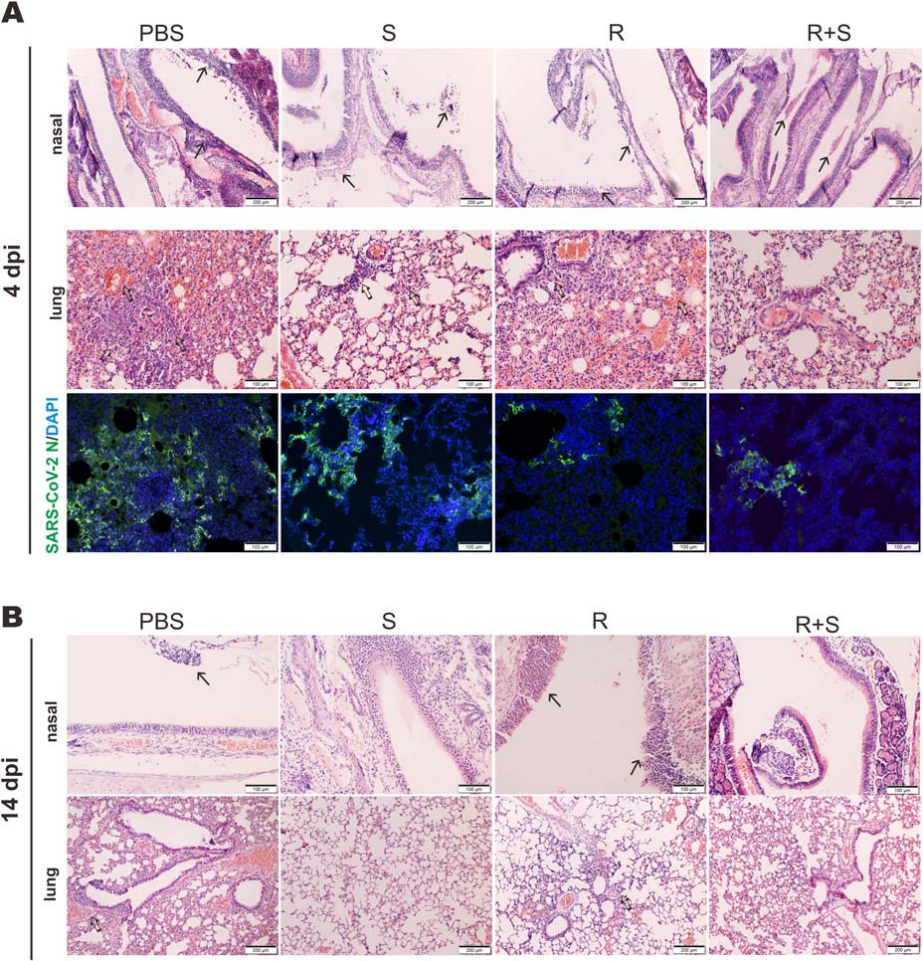
Effect of steroid and/or remdesivir on the histopathological changes in nasal turbinates and lungs of SARS-CoV-2-infected hamsters. Representative sections of the nasal turbinate and lung tissues from hamsters harvested at 4 dpi (A) and 14 dpi (B) were stained with hematoxylin and eosin. The arrows indicate inflammatory cell infiltration. Detection of SARS-CoV-2 N protein in lung tissue of SARS-CoV-2-infected hamsters treated with steroid (S) and/or remdesivir (R). SARS-CoV-2 N protein (green) was probed with rabbit anti-SARS-CoV-2 N antibodies followed by goat anti-rabbit antibodies conjugated to fluorescein. Nuclei were counterstained with DAPI (blue). Scale Bar = 100 μm for nasal turbinates; 200 μm for lungs.

**Figure 7.**
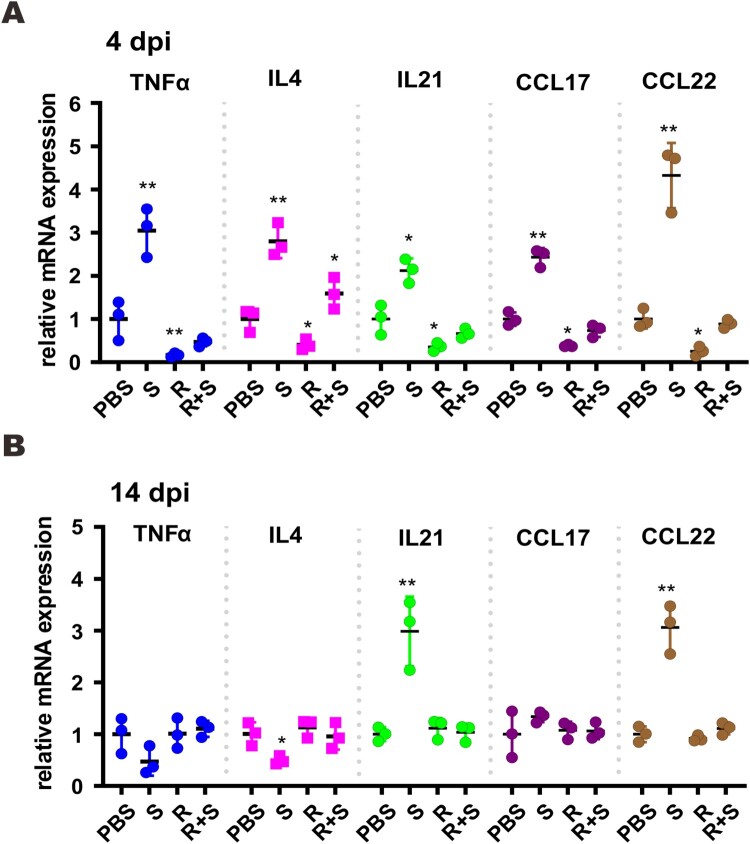
Proinflammatory cytokine and chemokine gene expression in SARS-CoV-2-infected hamster. Transcripts of representative chemokines and cytokines in the lung tissue homogenates of the indicated groups were detected by RT-qPCR at 4 dpi and 14 dpi. Results are shown as mean ± SD. Difference between the indicated groups was statistically significant as judged by Student’s t test (*P < 0.05; **P < 0.01). S: steroid only. R: remdesivir only. R + S: remdesivir plus steroid.

To eliminate the proviral effect of glucocorticoids that leads to reactivation of viruses such as hepatitis B virus, glucocorticoids are commonly prescribed in combination with antivirals [37]. With this in mind, we tested the effect of methylprednisolone plus remdesivir in SARS-CoV-2-infected hamsters. Methylprednisolone was given via intraperitoneal or intramuscular injection at 2 dpi. Remdesivir was administered intraperitoneally once daily at 2 dpi and 3 dpi (Figure 5(A)). Treatment with steroid alone (S), remdesivir alone (R), or remdesivir plus steroid (R + S) led to reversal of body weight loss with no noticeable difference (Figure 5(B)). However, viral RNA loads in the tracheas and lungs at 4 dpi as well as in the nasal turbinates, tracheas and lungs at 14 dpi decreased in hamsters treated with remdesivir or remdesivir plus methylprednisolone compared to counterparts receiving methylprednisolone alone, which increased above the background (Figure 5(C, D)). The antibody response to SARS-CoV-2 RBD was adversely affected in both the methylprednisolone and the remdesivir groups, but the decrease was much less severe in the remdesivir group compared to the methylprednisolone group (Figure 5(E)). It was more noteworthy that the significant drop in anti-RBD antibody response ascribed to methylprednisolone was partially rescued by remdesivir (Figure 5(E)). Whereas inflammatory cell filtration was still evident in nasal turbinate and lung tissues from hamsters treated with remdesivir alone, this was not seen in tissues from hamsters receiving remdesivir plus methylprednisolone (Figure 6(A, B)). Concurrent immunohistochemical staining of SARS-CoV-2 N protein in nasal turbinate and lung tissues indicated that the suppressive effect on N protein expression was more pronounced when hamsters were treated with remdesivir or remdesivir plus methylprednisolone (Figure 6(A) and S2). In contrast, N protein expression in methylprednisolone-treated cells was robust and not reduced compared to the background level (Figure 6(A)). When cytokine and chemokine expression was compared, we noted that the levels of all transcripts were close to basal in hamsters treated with remdesivir plus methylprednisolone (Figure 7(A, B)). Thus, a combination of methylprednisolone with remdesivir exhibited both antiviral and anti-inflammatory effect leading to the suppression of viral replication, inflammation and tissue damage.

## Discussion

There is an urgent need to develop safe and effective strategies for the treatment of COVID-19. In this study, we demonstrated that the use of methylprednisolone alone or in combination with remdesivir is beneficial to hamsters infected with SARS-CoV-2. In infected hamsters treated with methylprednisolone plus remdesivir, tissue inflammation was relieved and SARS-CoV-2 replication was inhibited. Thus, the combination of methylprednisolone and remdesivir for the treatment of COVID-19 merits further evaluation in randomized clinical trials.

Although SARS-CoV-2 replication and infection are the root cause of COVID-19, immune-mediated tissue damage and inflammation are the culprit in most severe and fatal cases [1,23]. Whereas early administration of antivirals that inhibit SARS-CoV-2 replication confers benefits to patients with COVID-19, anti-inflammatory treatment also proves crucial particularly in the late phase of infection and in severe and life-threatening cases [11]. Anti-inflammatory agents such as a humanized monoclonal antibody against IL6 receptor known as tocilizumab [38] and an IL1 receptor-antagonizing protein named anakinra [39] have been shown to be efficacious in the treatment of severe COVID-19. Nevertheless, simultaneous inhibition of both viral replication and pathologic inflammation should be more desirable.

The use of glucocorticoids including dexamethasone and methylprednisolone is more controversial due to their potential stimulatory effect on coronaviral replication [24–27]. A preliminary report from Britain indicated that treatment with dexamethasone confers survival benefits to patients with severe COVID-19 requiring respiratory support, but no alleviating effect was seen in patients with milder disease who did not require respiratory support [19]. In two other Brazilian studies, only hospitalized patients who were either suffering from moderate to severe acute respiratory distress syndrome or over 60 years of age had survival benefits after treatment with dexamethasone or methylprednisolone [20, 21]. Likewise, beneficial effects of corticosteroid were only seen in a subset of Chinese patients at the early phase of excessive inflammation [22]. It is not known whether inflammation is insignificant or non-responsive to glucocorticoid in patients with less severe COVID-19. Alternatively, the anti-inflammatory activity of glucocorticoid might be overshadowed by its proviral effect, particularly at the early stage of infection. At the late stage, the antibody response, which was shown to be affected adversely by methylprednisolone in our study, might fulfill the function to inhibit and eliminate SARS-CoV-2. The beneficial effect of methylprednisolone alone does indicate that viral replication might not be critical and could even be decoupled with inflammation and tissue damage at the later stage of infection. We demonstrated the stimulatory effect of methylprednisolone on viral RNA replication in human MDMs infected with two different doses of SARS-CoV-2. Plausibly, similar promotion might occur in macrophages and other cells *in vivo* under some circumstances. Further investigations are required to clarify when and where methylprednisolone might sensitize human cells to natural infection with SARS-CoV-2. The treatment of SARS-CoV-2-infected hamsters with methylprednisolone alone prevented body weight loss, improved tissue damage and inflammation, and suppressed the development of anti-RBD antibodies, which are thought to be neutralizing [40,41]. However, tissue viral RNA loads and viral titers increased immediately after treatment with methylprednisolone and viral RNA remained detectable at a later time point. The high viral loads could plausibly account at least in part for the high induction of proinflammatory cytokine and chemokine expression at the same time point. These phenotypes were largely reversed when a combination of methylprednisolone and remdesivir was used, suggesting that methylprednisolone plus remdesivir might be a safe and effective treatment for COVID-19. This principle of combining an effective antiviral with an anti-inflammatory agent should also be applicable to other drugs such as recombinant interferons and inhibitors of inflammasome.

The anti-RBD antibody response was found to be affected modestly in hamsters treated with remdesivir. This is generally consistent with the findings that the production of neutralizing antibodies was less robust in asymptomatic carriers of SARS-CoV-2 and patients with mild COVID-19 compared to those who develop a severe disease [42–44]. In contrast, the anti-RBD antibody response was much more severely compromised in hamsters treated with methylprednisolone alone, indicating its strong immunosuppressive effect [18]. In this regard, the reversal of this effect of methylprednisolone by remdesivir should be beneficial and would plausibly facilitate viral clearance and recovery from the disease.

The rise and fall of proinflammatory cytokines and chemokines in infected hamsters receiving different treatments are determined by multiple factors. Whereas infection with SARS-CoV-2 generally induces their expression leading to a cytokine storm [1,23,45], their levels were not consistently dampened by methylprednisolone under all circumstances. The suppressive effect on cytokine and chemokine expression was best seen at an early time point after delayed treatment with methylprednisolone. This might reflect the alleviation of inflammation. However, the levels of cytokines and chemokines were not decreased in hamsters receiving early steroid treatment or at a late time point, although alleviation of inflammation was evident upon histopathological examination. This pattern was not altered in the presence of remdesivir. Glucocorticoids fulfil their anti-inflammatory function at multiple levels, some of which might be downstream of the induction of proinflammatory cytokines [18,46]. Thus, the levels of proinflammatory cytokines and chemokines might not be good markers of tissue inflammation in animals treated with methylprednisolone or methylprednisolone plus remdesivir.

The dose, administration route and timing of glucocorticoid treatment in COVID-19 remain to be further optimized. Although single intravenous dose of pulse methylprednisolone has been shown to be beneficial to patients with severe COVID-19 [35], dexamethasone was given orally over 10 days in the larger randomized clinical trial in support of its use in British patients requiring oxygen or mechanical ventilation [19]. In the Brazilian trials, dexamethasone and methylprednisolone was injected intravenously for five days [20,21]. The treatment period of oral methylprednisolone for the Chinese patients was less than seven days [22]. In our study, the tissue viral loads in hamsters treated with a single intramuscular dose of methylprednisolone increased at 4 dpi and remained detectable at 14 dpi. It will be of interest to see whether viral replication might be boosted more significantly after use of glucocorticoid alone for 5 or 10 days and whether co-administration of remdesivir could sufficiently suppress SARS-CoV-2 in this context. Our results indicated that the antibody response was not affected by single dose pulse methylprednisolone therapy. As such, viral clearance would unlikely be delayed. Whether the same or similar pattern might be observed upon prolonged treatment with glucocorticoid for 5 or 10 days remains unclear. Both early and delayed treatments with methylprednisolone conferred benefits to hamsters infected with SARS-CoV-2 in our experimental setting. These results suggested that early and late intervention of inflammation might be equally important in the treatment of severe COVID-19. However, blockage of viral replication in the early phase of infection when immune response has not been fully developed should also be crucial. It remains to be seen whether combinational methylprednisolone and remdesivir might benefit more patients and have a more desirable safety and efficacy profile in future randomized controlled clinical trials.

## Supplementary Material

Suppl-2Jan.docx
