# Supplementary material for: Beneficial effect of combinational methylprednisolone and remdesivir in hamster model of SARS-CoV-2 infection
Source: Emerg Microbes Infect. 2021 Feb 25;10(1):291–304. doi: 10.1080/22221751.2021.1885998 (PMC7919885; doi:10.1080/22221751.2021.1885998)
Supplement: Suppl-2Jan.docx [file TEMI_A_1885998_SM1770.docx]

Supplementary data

**Beneficial effect of combinational methylprednisolone and remdesivir in hamster model of SARS-CoV-2 infection**

Zi-Wei Ye^a,b*^, Shuofeng Yuan^a,b*^, Jasper Fuk-Woo Chan^a,b*^, Anna Jinxia Zhang^a,b^, Ching-Yun Yu^c^, Chon Phin Ong^c^, Dong Yang^a^, Chris Chun-Yiu Chan^a^, Kaiming Tang^a^, Jianli Cao^a^, Vincent Kwok-Man Poon^a^, Chris Chung-Sing Chan^a^, Jian-Piao Cai^a^, Hin Chu^a,b^, Kwok-Yung Yuen^a,b^ and Dong-Yan Jin^b,c^

^a^State Key Laboratory of Emerging Infectious Diseases and Department of Microbiology, The University of Hong Kong, 122 Pokfulam Road, Pokfulam, Hong Kong

^b^Centre for Virology, Vaccinology and Therapeutics, Health@InnoHK, The University of Hong Kong, Pokfulam, Hong Kong

^c^School of Biomedical Sciences, The University of Hong Kong, 21 Sassoon Road, Pokfulam, Hong Kong

Supplementary data include 2 figures and 1 table.

**Figure S1** Effect of methylprednisolone on SARS-CoV-2 replication in monocyte-derived macrophages at 0.1 MOI.

**Figure S2** Analysis on the tissue damage and SARS-CoV-2 N protein expression in nasal turbinates and lungs of SARS-CoV-2-infected hamsters treated with steroid (S) and/or remdesivir (R) at 2 dpi.

**Table S1** Primers used in this study.


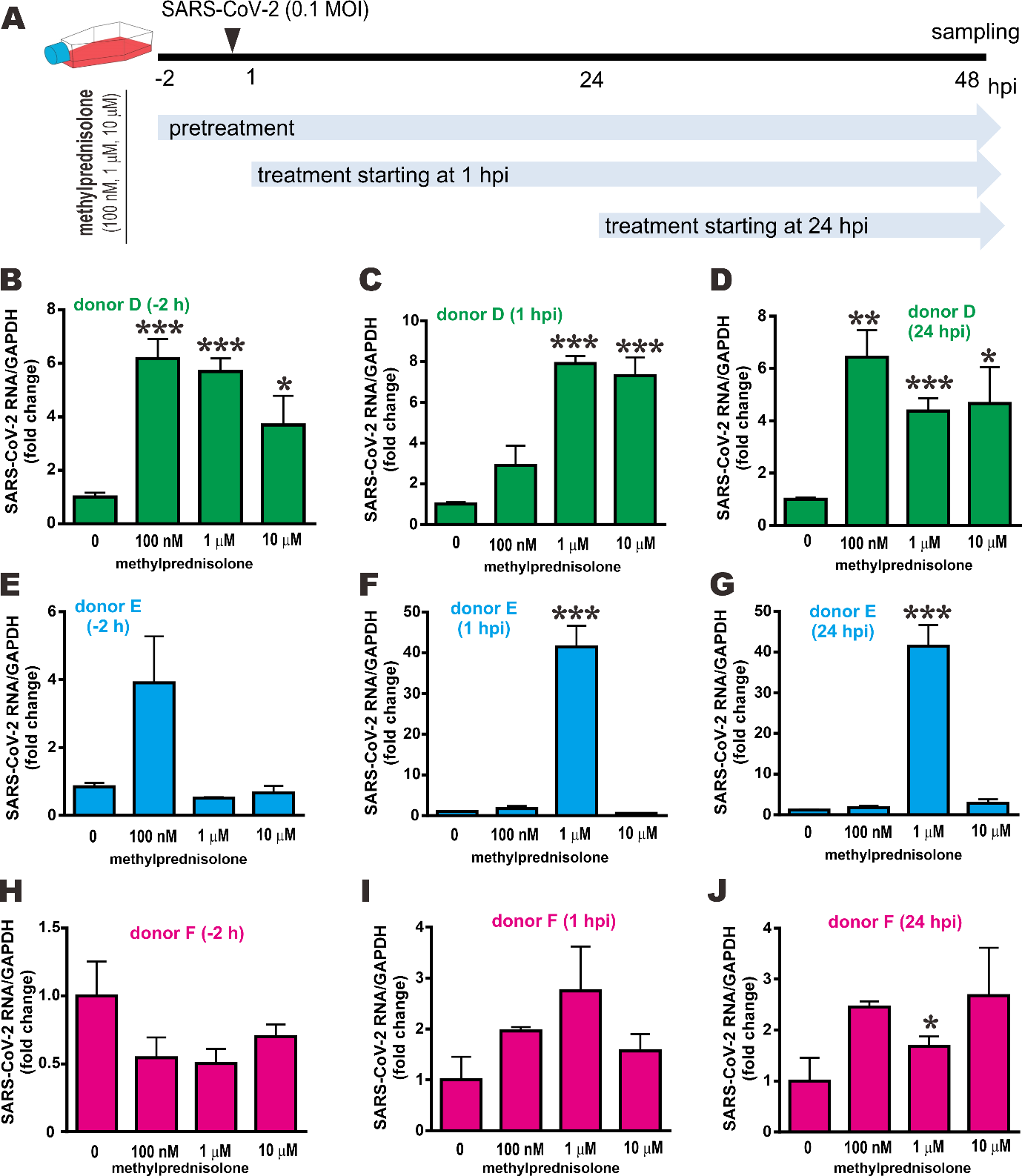


**Figure S1.** Effect of methylprednisolone on SARS-CoV-2 replication in monocyte-derived macrophages at 0.1 MOI. (A) Treatment scheme indicating different time points and doses of methylprednisolone addition before or after SARS-CoV-2 infection at 0.1 MOI in monocyte-derived macrophages from three donors. (B-J) Intracellular viral RNA loads. Cell lysates were collected at 48 hpi and viral genome copies were detected by RT-qPCR. Results are presented as fold change after normalization to human GAPDH transcript. Difference between the indicated group and the no treatment group was statistically significant as judged by Student’s t test (*: P < 0.05; **: P < 0.01; ***: P < 0.001).

**DAPI**

**SARS-CoV-2 N**

**SARS-CoV-2 N/DAPI**

A

B


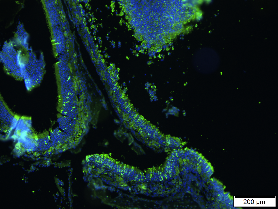

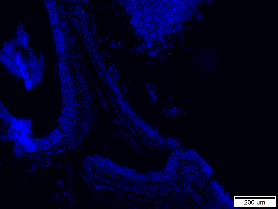

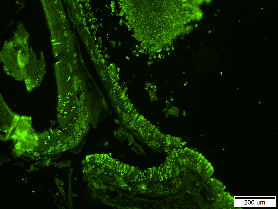

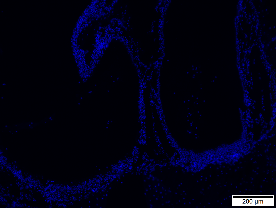

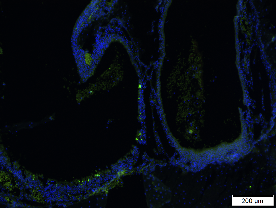

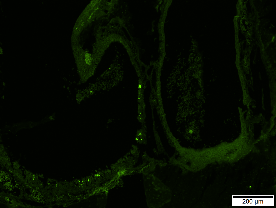

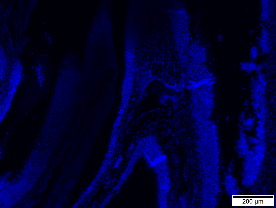

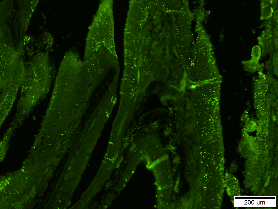

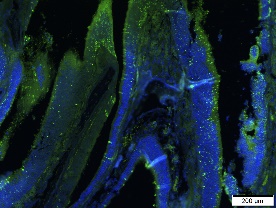

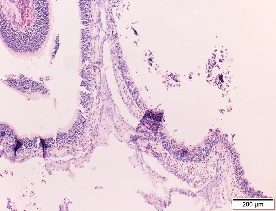

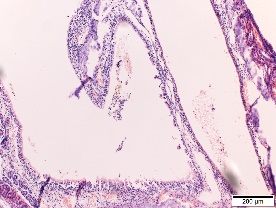

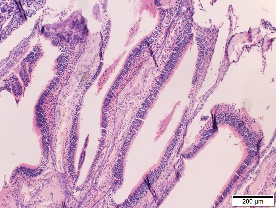


**H & E**

**S**

**R**

**R+S**


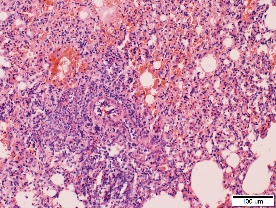

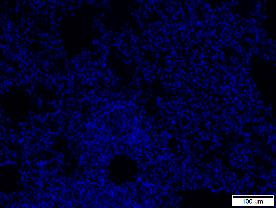

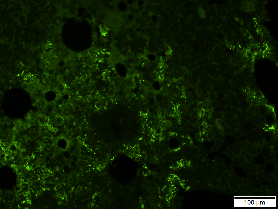

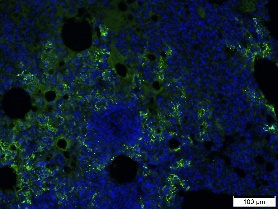


**DAPI**

**SARS-CoV-2 N**

**SARS-CoV-2 N/DAPI**

**H & E**

**S**


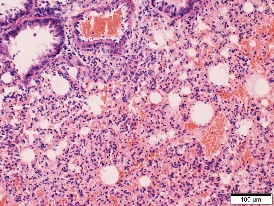

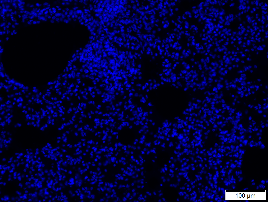

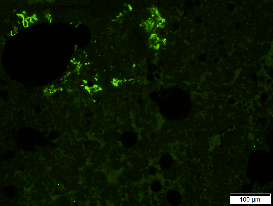

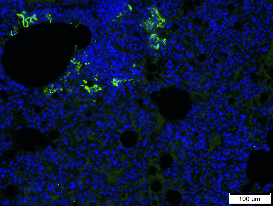

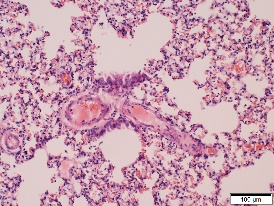

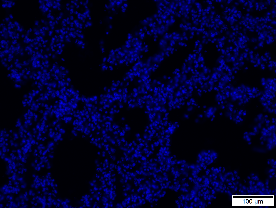

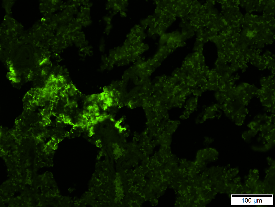

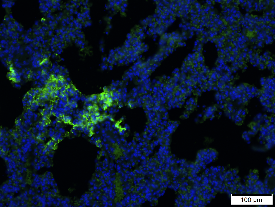


**R**

**R+S**

**Figure S2.** Tissue damage and SARS-CoV-2 N protein expression in nasal turbinates (A) and lungs (B) of SARS-CoV-2-infected hamsters treated with steroid (S) and/or remdesivir (R) at 2 dpi. Samples were harvested at 4 dpi. SARS-CoV-2 N protein (green) was probed with rabbit anti-SARS-CoV-2 N antibodies followed by goat anti-rabbit antibodies conjugated to fluorescein. Nuclei were counterstained with DAPI (blue). Scale Bar = 100 μm for nasal turbinates; 200 μm for lungs.

**TABLE S1 Primers used in this study.**

| Name | Sequence (5′ – 3′) |
| --- | --- |
| Human GAPDH forward | GTCTCCTCTGACTTCAACAGCG |
| Human GAPDH reverse | ACCACCCTGTTGCTGTAGCCAA |
| Human β-tubulin forward | GGACGAGATGGAGTTCACCG |
| Human β-tubulin reverse | GAGGAAAGGGGCAGTTGAGT |
| Human IL6 forward | AGACAGCCACTCACCTCTTCAG |
| Human IL6 reverse | TTCTGCCAGTGCCTCTTTGCTG |
| Human CXCL10 forward | TCTCCGAGATGCCTTCAGCAGA |
| Human CXCL10 reverse | TCAGACAAG GCTTGGCAA CCCA |
| Hamster β-actin forward | ACTGCCGCATCCTCTTCCT |
| Hamster β-actin reverse | TCGTTGCCAATGGTGATGAC |
| Hamster γ-actin forward | ACAGAGAGAAGATGACGCAGATAATG |
| Hamster γ-actin forward | GCCTGA ATGGCCACGTACA |
| Hamster TNFα forward | TGAGCCATCGTGCCAATG |
| Hamster TNFα reverse | AGCCCGTCTGCTGGTATCAC |
| Hamster IL4 forward | ACAGAAAAAGGGACACCATGCA |
| Hamster IL4 reverse | GAAGCCCTGCAGATGAGGTCT |
| Hamster IL21 forward | GGACAGTGGCCCATAAAACAAG |
| Hamster IL21 reverse | TTCAACACTGTCTATAAGATGACGAAGTC |
| Hamster CCL17 forward | GTGCTGCCTGGAGATCTTCA |
| Hamster CCL17 reverse | TGGCATCCCTGGGACACT |
| Hamster CCL22 forward | TGGTGCCAA CGTGGAAGAC |
| Hamster CCL22 reverse | GAAGAACTCCTTCACTACGCGC |
| SARS-CoV-2 RDRP forward | CGCATACAGTCTTRCAGGCT- |
| SARS-CoV-2 RDRP reverse | GTGTGATGTTGAWATGACATGGTC |
| SARS-CoV-2 RDRP probe | FAM-TTAAGATGTGGTGCTTGCATACGTAGAC-lABkFQ |
